# Carbon supported ternary layered double hydroxide nanocomposite for Fluoxetine removal and subsequent utilization of spent adsorbent as antidepressant

**DOI:** 10.1038/s41598-024-53781-y

**Published:** 2024-02-17

**Authors:** Samar M. Mahgoub, Doaa Essam, Zienab E. Eldin, S. A. Abdel Moaty, Mohamed R. Shehata, Ahmed Farghali, Saif Elden B. Abdalla, Sarah I. Othman, Ahmed A. Allam, Fatma I. Abo El-Ela, Rehab Mahmoud

**Affiliations:** 1 Materials Science and Nanotechnology Department, Faculty of Postgraduate Studies for Advanced Sciences, Beni-Suef University, Beni-Suef, Egypt; 2 Nanomaterials Science Research Laboratory, Chemistry Department, Faculty of Science, Beni-Suef University, Beni-Suef, Egypt; 3 Department of Chemistry, Faculty of Science, Beni-Suef University, Beni-Suef, Egypt; 4 Chemistry Department, Faculty of Science, Cairo University, Giza, Egypt; 5 Department of Medical Laboratory Science. College of Applied Medical Science, Jazan University, Jazan, Saudi Arabia; 6 Department of Biology, College of Science, Princess Nourah Bint Abdulrahman University, P.O. BOX 84428, 11671 Riyadh, Saudi Arabia; 7 Department of Biology, College of Science, Imam Muhammad Ibn Saud Islamic University, 11623 Riyadh, Saudi Arabia; 8 Department of Zoology, Faculty of Science, Beni-Suef University, 62511 Beni-Suef, Egypt; 9 Department of Pharmacology, Faculty of Veterinary Medicine, Beni-Suef University, Beni-Suef, Egypt

**Keywords:** Fluoxetine, Wastewater, Adsorption, Spent adsorbent, Toxicity, Antidepressant, Drug delivery, Nanobiotechnology, Environmental chemistry

## Abstract

Fluoxetine (FLX) is one of the most persistent pharmaceuticals found in wastewater due to increased use of antidepressant drugs in recent decades. In this study, a nanocomposite of ternary ZnCoAl layered double hydroxide supported on activated carbon (LAC) was used as an adsorbent for FLX in wastewater effluents. The nanocomposite was characterized using Fourier Transform Infrared Spectroscopy (FTIR), scanning electron microscope (SEM), transmission electron microscope (TEM), X-ray diffraction (XRD), and surface area analysis (BET). The adsorption investigations showed that the maximum removal capacity was achieved at pH 10, with a 0.1 g/L adsorbent dose, 50 mL volume of solution, and at a temperature of 25 °C. The FLX adsorption process followed the Langmuir–Freundlich model with a maximum adsorption capacity of 450.92 mg/g at FLX concentration of 50 µg/mL. Density functional theory (DFT) computations were used to study the adsorption mechanism of FLX and its protonated species. The safety and toxicity of the nanocomposite formed from the adsorption of FLX onto LAC (FLX-LAC) was investigated in male albino rats. Acute toxicity was evaluated using probit analysis after 2, 6, and 24 h to determine LD_50_ and LD_100_ values in a rat model. The FLX-LAC (20 mg/kg) significantly increased and lengthened the sleep time of the rats, which is important, especially with commonly used antidepressants, compared to the pure standard FLX (7 mg/kg), regular thiopental sodium medicine (30 mg/kg), and LAC alone (9 mg/kg). This study demonstrated the safety and longer sleeping duration in insomniac patients after single-dose therapy with FLX-LAC. Selective serotonin reuptake inhibitors (SSRIs) like FLX were found to have decreased side effects and were considered the first-line mood disorder therapies.

## Introduction

**Figure 1:**
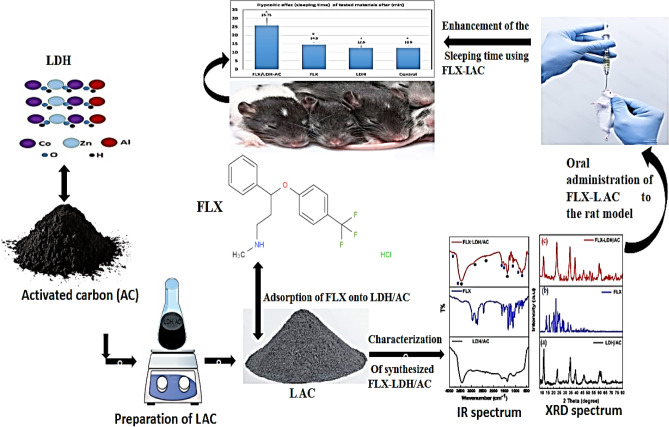
The procedure of synthesis of the nanocomposite illustrating its significant role in Sleep promotion in rat model as a spent adsorbent.

Depression is a common feature of modern life, often caused by stress and leading to high use of antidepressants. One such antidepressant is Fluoxetine HCL (FLX), which is the most widely used worldwide. Studies have found that about 70% of FLX doses are excreted in urine and feces, leading to high levels of FLX in wastewater^1^. This can have negative effects on the environment, as well as on animal and human health, even at low concentrations^2,3^. FLX and its byproducts are frequently present in water environments at levels of up to 22 μg L^−1^^4^. FLX was found in high concentrations in influents and effluents at 3.4 and 2.7 g/L in Asia–Pacific, Europe, and North America^5^. The Risk Quotient (RQ) for FLX indicates a medium to high risk to aquatic species, with a calculated RQ of 1.51^4,5^. Several attempts to remove FLX from water were unsuccessful because they did not completely remove the FLX^6,7^. Several technologies are generally available for removal of pharmaceuticals from wastewater streams such as advanced oxidation process, heterogeneous photocatalysis, ozonation, electrochemical oxidation, membrane treatment, and adsorption^8^. Adsorption can be defined as the net accumulation of a chemical species at the interface between a solid phase and an aqueous solution phase^9^. This process is effective for removal of cationic, anionic, or organic pollutants from wastewater streams by uptaking such pollutants on the external surface of adsorbent by complexation or ionic exchange mechanism^10^. Adsorption is a verstaile and cost-effective process for removing pollutants from wastewater that requires low initial investment and simple adsorber design while showing operational simplicity and high pollutant removal effeciency^11^. In addition, adsorption is suitable for low adsorbate concentrations, suitable for batch or continuous operations, low consuming energy process, and adsorbents can be regenerated and reused^12^. All such advantages led to adaptation of adsorption for pharmaceutical and other pollutants removal with a wide investigation of adorbents that show high effeciecny and low cost. Studies have shown that waste-based biosorbents such as granular activated carbon (GAC), synthetic zeolites, and materials like spent coffee grounds (SCG), pine bark, and cork waste can effectively remove pollutants like FLX^13^. Earlier studies found that the maximum adsorption capacities were 6.41 mg/g and 5.076 mg/g when using biochars made from forest and agri-food waste, and cross-linked β-cyclodextrin carboxy methylcellulose polymer, respectively^14,15^. The adsorption capacities using Phycoremediation and adsorbents produced from paper mill sludge were 2.1 mg/g and 191.6 mg/g, respectively^16,17^. This study involved creating a more effective adsorbent by combining a ternary layered double hydroxide (LDH) with activated carbon to remove FLX from wastewater. In addition to adsorption, we also focused on finding a solution for the disposal of used adsorbents. FLX is widely used for treating depression, sleep disorders, panic attacks, bulimia nervosa, and obsessive–compulsive disorder^18^. We studied the use of spent adsorbent as a drug carrier to assess the release of FLX, an important drug for extending the effects of sleep or as a hypnotic drug. Sustained release of FLX is necessary to address challenges such as prolonged time to fall asleep, increased number and duration of awakenings during sleep, decreased sleep efficiency, and early morning awakenings,which are signs of disrupted sleep for patients with depression^19–21^. A new FLX formulation is being studied for treating sleep disorders and depression. The study will investigate a nanocomposite as an adsorbent for FLX removal and assess its performance as an antidepressant in a sleep model. See Fig. 1 for more details.

## Experimental work

### Materials

We obtained Cobalt Chloride hexahydrate (CoCl_2_⋅6H_2_O), Zinc Chloride (ZnCl_2_), Aluminum Chloride (AlCl_3_), Activated carbon, Sodium Hydroxide, Methanol, Stabilizer free tetrahydrofuran, Phosphoric acid, Triethylamine, and 99.8% pure Ethanol from Sigma-Aldrich. Hydrochloric acid of HPLC analytical grade was obtained from Scharlau. All preparations were made using Milli-Q water with a resistivity of 18.2 MΩ cm. Monobasic Potassium Phosphate was obtained from Merck, and FLX HCL (C17H19ClF3NO, CAS No. 56296-78-7, Molecular weight: 345.78) was obtained from Shandong Octagon Chemicals Limited in China.

### Synthesis of ZnCoAl LAC (LAC)

ZnCoAl LDH was prepared using a simple co-precipitation method with a Zn:Co:Al molar ratio of 2:2:1. The metal salts were dissolved in 500 mL of distilled water with 1.00 g AC and shaken overnight. A 2 M NaOH solution was added dropwise until the pH reached 9.00 at 60 °C. The suspension was stirred for 24 h, then the precipitate was separated by centrifugation and washed with bi-distilled H_2_O to remove any remaining NaOH (pH = 7). Finally, the precipitate was dried at 80 °C for 12 h and ground to a uniform particle size^22–24^.

### Material characterization

The LAC was characterized using various methods. X-ray diffractometer with Cu-Kα radiationwas used to determine crystallinity, Fourier transform infrared spectroscopy was used to identify functional groups, and Field Emission Scanning Electron Microscope was used to investigate morphology. The residual FLX concentrations were determined using an Agilent 1200 HPLC system. The pH of the solution was measured using a pH meter, and hydrodynamic particle sizes and zeta potential were measured using Malvern zetasizer equipment. The surface area, pore volume, and pore diameters were measured using nitrogen adsorption–desorption isotherm. Thermal stability and phase transition were investigated using TGA/DTA instrument.

### Adsorption of FLX study

Diluted concentrations ranging from 5 to 500 µg/mL were prepared from a stock standard solution of 1000 µg/mL to create a calibration curve at room temperature. To study the effect of pH on the adsorption process, six 50 mL falcon tubes were prepared with 0.05 g of synthetic adsorbent (LAC) and 50 µg/mL FLX. The pH of the solutions was adjusted to pHs of 5, 7, 9, 10, and 11 using 0.1 N NaOH or 0.1 N HCl and placed on an orbital shaker for 24 h at 250 rpm. The same steps were repeated in six tubes without the adsorbent. The effect of the dose of adsorbent on the adsorption process was examined at a constant FLX concentration of 50 µg/mL using doses ranging from 0.0125 to 0.20 g. The effect of FLX concentration was also examined using concentrations ranging from 5 to 500 µg/mL. The thermal effect was examined at various temperatures: 15, 25, 35, 45, and 55 °C. The solutions were filtered using a Millipore Nylon 0.22 mm pore size syringe filter before measurements were taken. The determination and quantitation of FLX were carried out under isocratic conditions using a Nova-pak C8 column (3.9 × 150 mm, 4 m) and a mobile phase consisting of 600 mL of a buffer solution, 300 mL of stabilizer tetrahydrofuran, and 100 mL of methanol. The buffer solution was prepared by adding 10 mL of Triethylamine to about 980 mL of water and adjusting the pH to 6 with phosphoric acid. The analytical column was held at room temperature, and the injection volume and mobile phase flow rate were 10 µL and 1.0 mL/min, respectively.

The amount of the adsorbed FLX per grams and the removal percentage of LAC (Qe) were determined Using the following equations:

1 $$\text{Qe }= \frac{\left({{\text{C}}}_{{\text{o}}}-{\text{ C}}_{{\text{t}}}\right){\text{V}}}{{\text{W}}}$$

2 $$\text{Removal percent }= \frac{{{\text{C}}}_{{\text{o}}}-{\text{ C}}_{{\text{t}}} }{{{\text{C}}}_{{\text{o}}}} \times 100$$

The amount of adsorbed FLX per gram (Qe) is calculated using the initial (Co) and after adsorption (Ct) concentrations of FLX in mg/L at time T. V represents the volume of FLX, and W is the mass of the adsorbent in grams. Various isotherm models, including two, three, and four parameter models, were used. Thermodynamic parameters were also determined. Additionally, different kinetic models, such as pseudo-first-order kinetics^25^, Pseudo- Second-Order^26^, diffusion of intraparticles^27^ and Avrami^28^ at various time points between 0 and 240 min have been investigated to study the kinetics of FLX adsorption onto LAC/AC.

The Point of Zero Charge (PZC) of the LAC was determined by adding 0.05 g of thesynthesized LDH to 25 mL of aqueous solution at varying pH levels (5, 7, 9, 10, and 11). The solution was then allowed to sit for 24 h to reach the final pH. The difference between the finaland initial pH was plotted against the initial pH. The PZC is the initial pH at which the ∆pH = 0.

### DFT study

We used the Gaussian09 program with density functional theory (DFT) at the B3LYP/6-311G (d, p) theory level to determine the lowest energy geometries of Fluoxetine HCl and its protonated forms.

### Sustainable trends of using waste adsorbent

**Scheme 1:**
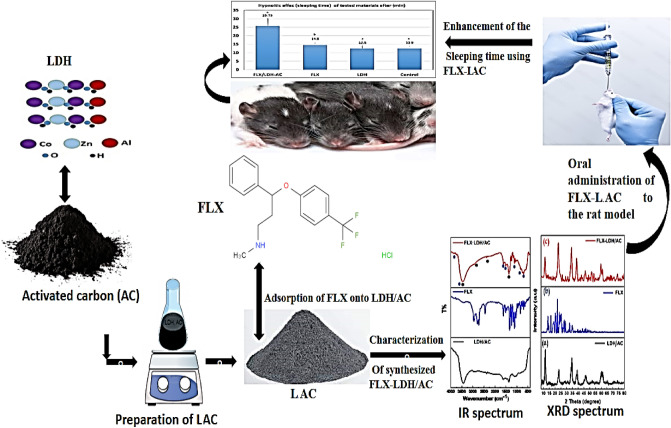
The procedure of synthesis of the nanocomposite illustrating its significant role in Sleep promotion in rat model as a spent adsorbent.

The waste adsorbent was collected and washed multiple times with bidistilled water. It was then dried in an oven at 50 °C for 24 h. Scheme 1 illustrates the synthesis procedure of the nanocomposite and its significant role in promoting sleep in a rat model.

### In-vivo search experimental animals

The Department of Physiology at Beni Suef University's Faculty of Veterinary Medicine purchased laboratory animals. The rats were housed in standard laboratory conditions at 23 °C, 60% humidity, and a 12-h light/dark cycle. Animal handling techniques, including weighing and gavage, were conducted in accordance with the Animal Rights Protocol for Laboratory Experiments approved by the Institutional Animal Care and Use Committee (IACUC) of Beni Suef University's Faculty of Science. Toxicity tests were conducted on adult rats weighing 160–180 g to determine the LD50 ratios of the FLX-LAC, FLX, and LAC nanocomposite. Rats used in acute studies had access to food and water 24 h a day.

#### Experimental groups and medications for to estimate the sleep time

40 mature male albino rats, with an average body weight of 150–250 g and aged 3–4 months, were divided into four equal groups for the study. Group 1 (G1) received 0.5 mL of distilled water orally as the negative control (CNT). Group 2 (G2) was given FLX-LAC Nano composite at a dose of 20 mg/kg body weight, while Group 3 (G3) received fluoxetine (FLX) alone at 7 mg/kg, and Group 4 (G4) received LAC at 9 mg/kg. All rats fell asleep 1 h after the sleep time test began. The doses of the tested drugs were calculated starting at 1/20 of the expected LD50.

#### The evaluation of LD50 and LD90 as toxicity indicators (probit analysis)

Converting the value evaluation of the mathematical model that best matches the dependent variable Y (percentage) into probit values allows the independent variable X to submit experimental data^29^. Experimental outcomes (X—different concentrations), regression, and probit statistics are superior to standard LD_50_ calculation methods, which include (percentage) at predetermined doses of investigated components and Y—death of experimental animals (regression analysis). Using data and interpolation, the number is calculated. the Miller-Tainter method^30^ enhances the experimental data variable Y by 50%^31^. If the mortality rate at the lower and/or higher doses is 0% and 100%, respectively, the Miller-Tainter method similarly changes the mortality outcomes (in percentage) into probit values, but first the percentage values are corrected against the number of experimental animals. these corrected values are converted into probit values. for further processing instead of % doses, the mg/kg doses were used to estimate LD_50_ and LD_90_.

#### Calculation of the maximum lethal dosage (LD100) and median lethal dose (LD50) for the nanomaterial under study

In this study, divided into 3 groups of 10 rats each. The rats were given doses of 50, 100, 150, 200, 250, 300, 350, 400, 450, 500, 550 and 600 mg/kg orally. The animals were monitored 2, 6 and 24 h after treatment and the mortality rate was calculated after 24 h. the miller-Interactive Tainter's LD_50_ estimation method was used to examine. rats with LD_0_, LD_20_, LD_50_, LD_90_ and LD_100_ values. A linear correlation coefficient was calculated using SPSS probability analysis to assess mortality trends with respect to the concentrations of the tested medications^32^.

#### Mortality and toxic symptoms

We observed mortality, physical characteristics, and behavior (such as drowsiness, salivation, and lethargy), as well as any injury or illness at 2, 6, and 24 h after injection.

#### Estimation of the hypnotic effect or sleep time impact of FLX-LDH/AC, FLX and LAC

Forty male rats were divided into four groups (each with ten rats): control group (untreated negative), FLX-LAC, FLX, and LAC (19.3, 7, 9 mg/kg b.wt.) respectively. One hour later, all groups received 30 mg/kg sodium thiopental intraperitoneally. Sleep time^33^ was measured by counting the time from the onset of unconsciousness until the rat woke up again for each group and for each rat.

### Statistical analysis

The mean and standard deviation (S.E.M.) were provided. Statistical significance was confirmed by Snedecor's one-way ANOVA, SPSS (version 20.0), and Tukey's post hoc test for multiple comparisons (IBM SPSS Statistic 20.0, Armonk, NY, USA). P values less than 0.05 were significant^34^.

### Ethical approval

All animals handling, including feeding, watering, dosing and other treatments were subjected to ARRIVE guidelines and IACUC of Beni-Suef University for animals caring and use committed for ethical treatment of lab animals in research and the committee approved the protocol of study.

## Results and discussion

### Adsorbent characterization

Figure 1 displays the X-ray diffraction patterns of LAC before and after FLX adsorption, LAC-FLX. The peaks at 11.42°, 23.41°, 34.49°, 38.99°, 46.60°, 60.09°, 61.2°, and 65.13° correspond to LDH peaks of (003), (006), (012), (015), (018), (110), (113), and (116)^35^. The AC material exhibited peaks at 26.41° and 63.8°, which are associated with the (002) and (221) indices^36^. The XRD result allowed for the identification of the synthesised AC's crystalline phase (Fig. 1). The formation of crystalline structures of graphitic carbon was confirmed by the diffraction patterns, which agree well with the standard JCPDS file (41-1487). The crystalline structure of AC has been verified by the presence of a sharp peak around 26.4O. Further spectroscopic analysis supported the XRD results, which showed that AC has a better crystallographic structure and well-organized aromatic carbon that is more stable than amorphous-like carbon^37^.

As shown in Fig. 1 the LAC sample similler to XRD patterns of LDH which could be attributed to the LDH nanoparticles' excellent formation and effective dispersion within the AC matrix structure^38^. Also, LAC displaying diffraction peaks that are symmetrical and sharp indicating that each of these carbon-coated LDH has a well-crystalized structure^39^.

**Figure 2:**
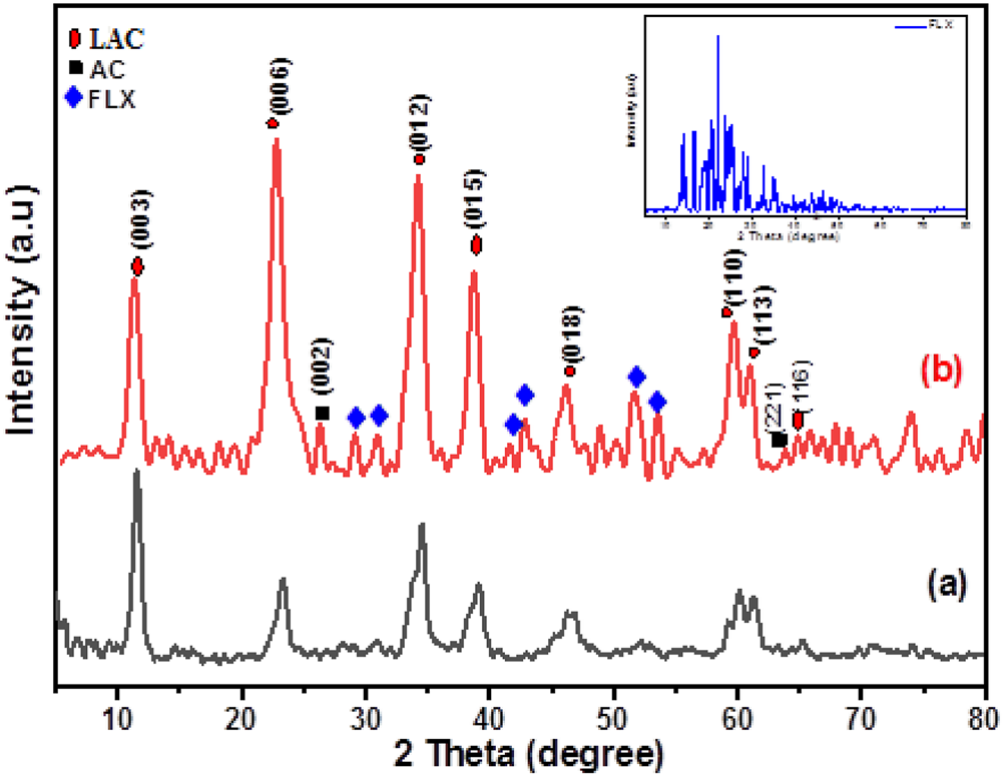
XRD of (**a**) LAC and (**b**) FLX-LAC, the Inset figure is the XRD of FLX.

The XRD patterns of FLX-LAC after FLX adsorption revealed peaks at 29.1°, 31°, 41.6°, 42.7°, 51.8°, and 53.5°, matching those of pure fluoxetine which was illustrated in inset Fig. 2.

**Figure 3:**
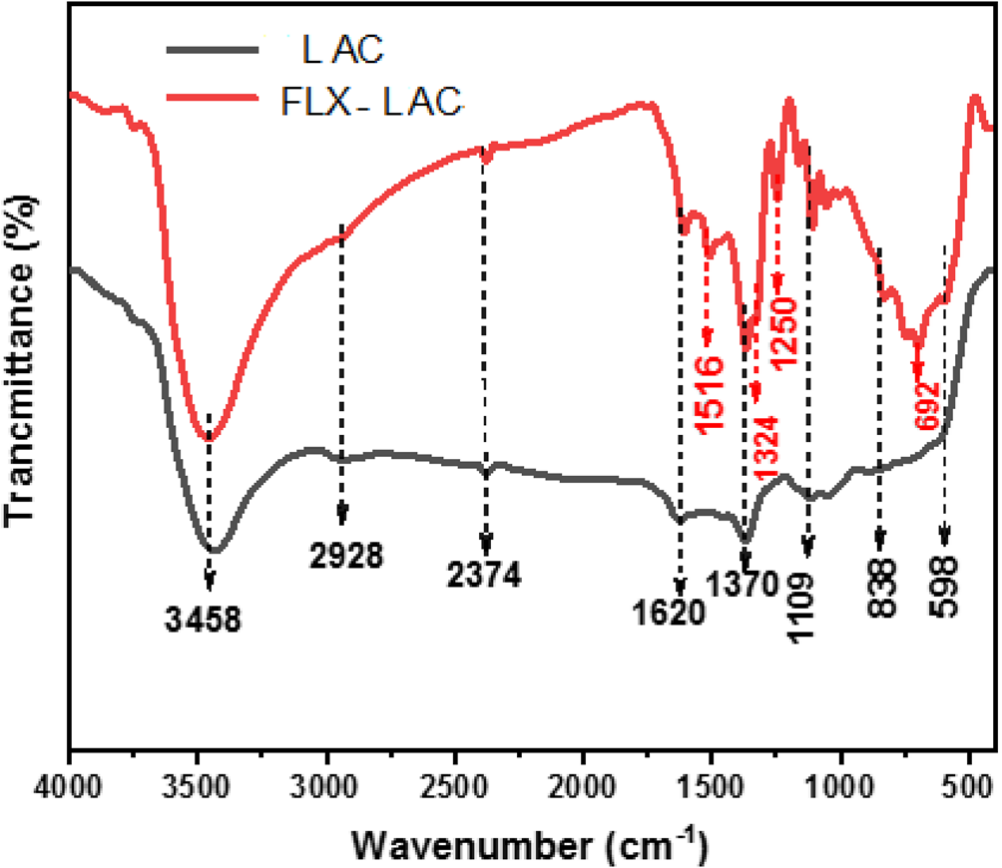
FTIR Spectrum of the prepared LAC and FLX-LAC.

The samples were analyzed using FT-IR spectroscopy to demonstrate the interaction mechanism and molecular structures, as shown in Fig. 3. The LAC spectrum showed a strong broad peak at 3458 cm^−1^, attributed to O–H stretching vibrations from water molecules and interlayer hydrogen bond^36^. The O–H bending vibration appeared at 1620 cm^−1^^40^. The stretching vibration of atmospheric CO_2_ caused the peak at 2374 cm^−1^^41^. Additionally, the peak at 2928 cm^−1^ originated from the C–H stretching vibration of the AC^36^. The LAC spectrum also had a clear peak at 1370 cm^−1^, explained by the presence of nitrate (NO3) groups between the LDH inter-layers^42^. The absorption peak at 1109 cm^−1^ was caused by the C–O stretching modes of AC. The bands at 838 cm^−1^, and 598 cm^−1^ were for M–O and M–O–H^43^.

**Figure 4:**
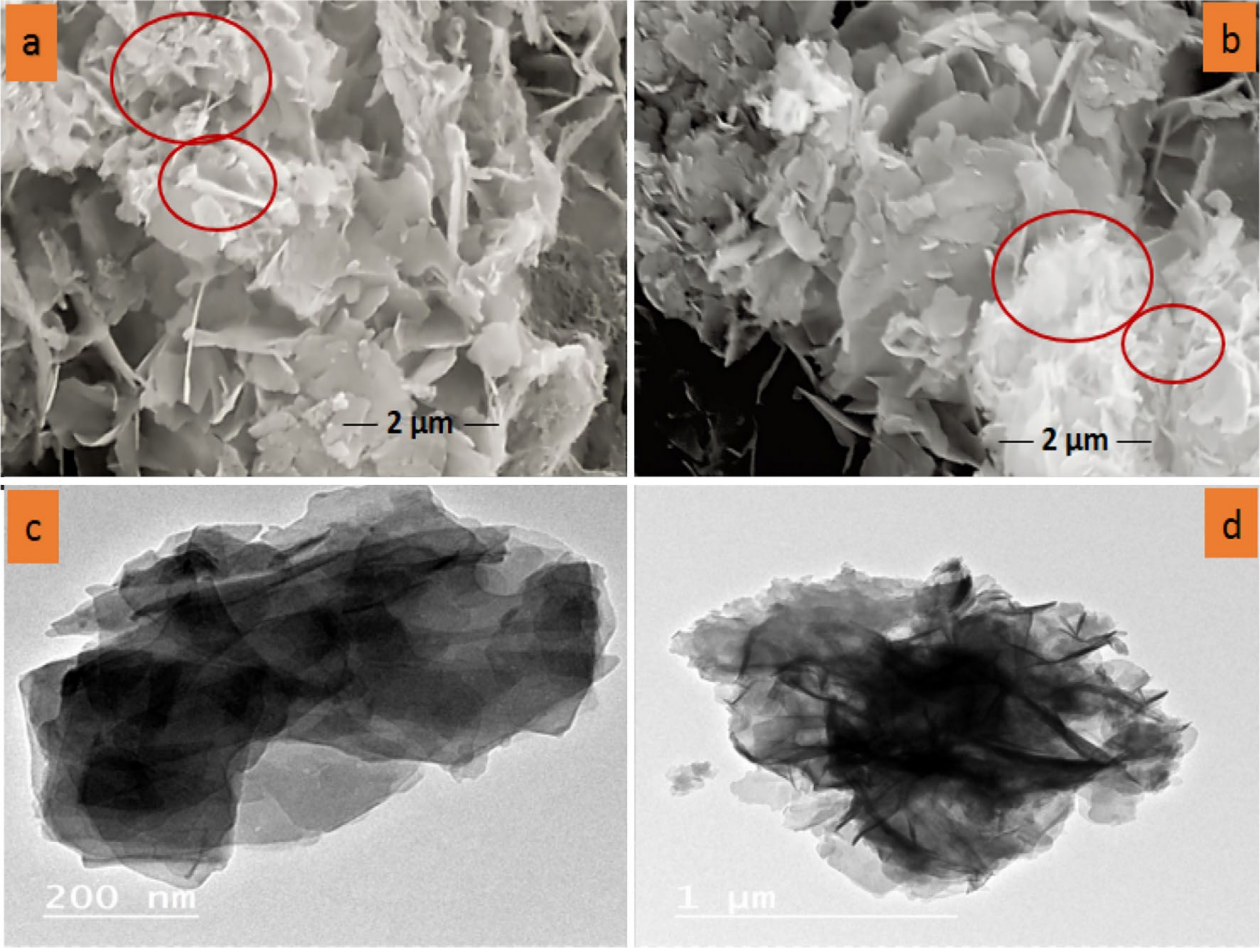
SEM (**a**, **b**) and TEM images of LAC (**c**, **d**).

The SEM micrographs in Fig. 4a,b show layers of irregular LDH grows with carbon closely and that appeared as a flower-like morphology of the LAC^44^, attributed to the presence of activated carbon. This structure has high porosity and homogeneity, resulting in a significantly larger surface area and higher adsorption capacity^45–47^. The HRTEM microscopy images in Fig. 4c,d show the incorporation of activated carbon within the LDH layers. In order to further illustrate the interaction behaviour of AC loading within the layers of LDH, HRTEM analysis of the LAC composites was carried out. The formation of a layer structure of LDH was visible in the HRTEM image of the LAC composite (Fig. 4c,d), and the random distribution of AC within the surface of LDH as shaded layer on LDH, indicated the composite's heterogeneous surface morphology. The proper content of AC and the coprecipitation synthesis method enable better and more efficient intercalation of AC into LDH layers. This enhanced the LAC composite's surface, structure, and textural properties, as demonstrated by the FT-IR, XRD, and BET analyses that are covered below.

**Figure 5:**
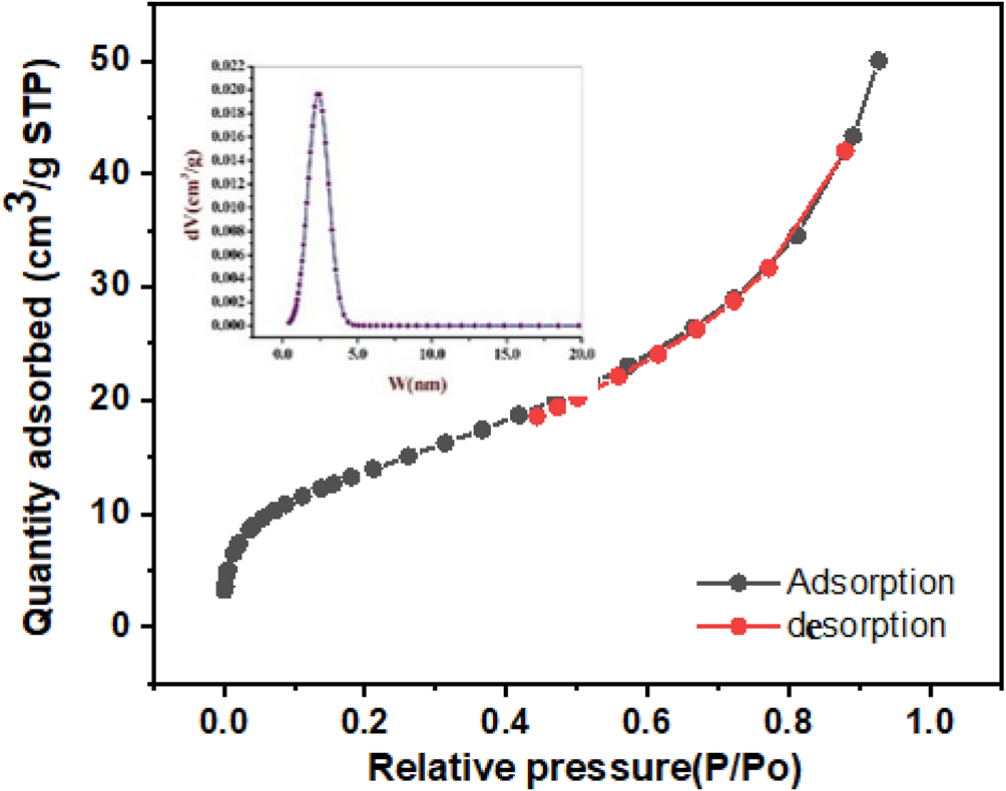
N_2_ sorption isotherms of LAC and inset is the NLDFT distributions.

According to the IUPAC classification system for adsorption isotherms, the N2 Adsorption–desorption isotherms of the LAC sample are classified as type IV, as shown in Fig. 5. The isotherms exhibit type H3-type hysteresis loops, indicating the presence of mesoporous structures resulting in a slit-shaped porous network structure that has been elucidated using SEM and TEM structures of LAC^24^. Table 1 clearly shows the experimental values of the LAC surface structure parameters, indicating that the prepared material has good surface texture.

**Table 1:** Parameters of Surface textures of the LAC nanocomposite.

Surface structure parameters	LAC
Specific surface area (m^2^/g)	303.79 m^2^/g
pore volume (cm^3^/g)	0.246 cm^3^/g
The monolayer capacity V_M_	69.80 cm^3^ (STP)/g
Average pore diameter (nm)	3.246 nm

### Adsorption analysis

#### The effect of pH on the adsorption process

**Figure 6:**
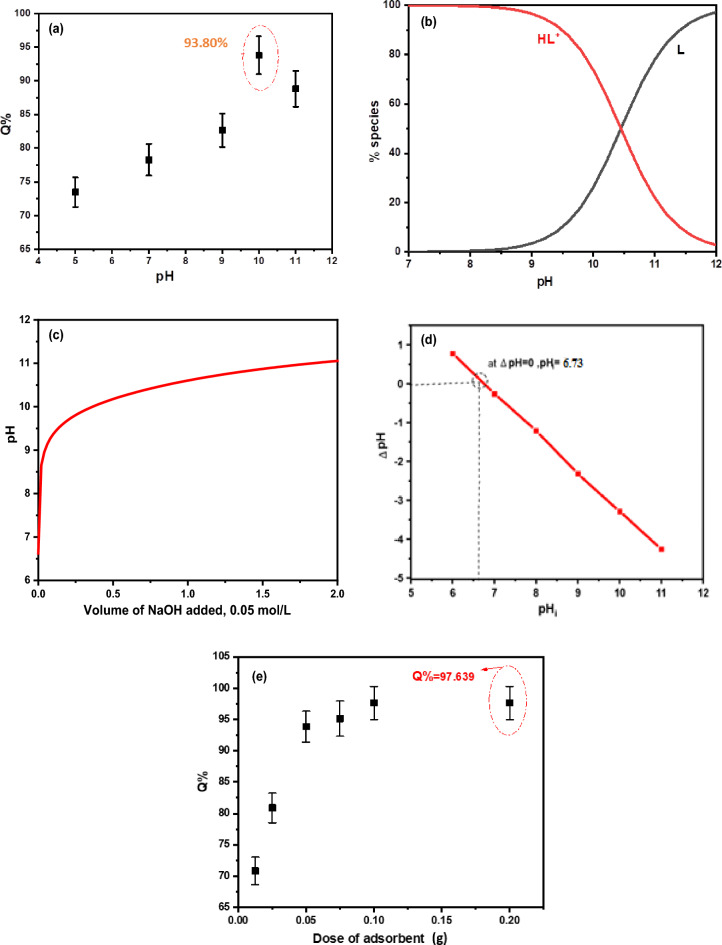
(**a**) removal efficiency of FLX (50 µg/mL) by LAC (0.05 g/50 mL) at various pH (**b**) distribution curve of the FLX at various pH (**c**) aqueous solution potentiometric titration curve of FLX (**d**) PZC of LAC and (**e**) The effect of Adsorbent Dose (LAC) on the FLX adsorption process (**d**).

The pH value is a crucial factor in the adsorption process, controlling the surface properties of both the adsorbent and the drug (Fig. 6a)^48^. The pH investigation covered a range from 5 to 11 showing stability within this range^49^. Higher pH values over 10 were excluded from the investigation due to the potential for structural disorder caused by metal hydroxides. The removal efficiency of LAC for the drug was highest at pH 10, reaching 85.15%. This is likely due to the point of zero charge (PZC) value of the LAC, which is 6.73 (Fig. 6d).

The species distribution curve for FLX species is shown in Fig. 6b, and the titration curve is given in Fig. 6c. Figure 6c from the data of potentiometric titration curve we calculate the dissociation constant of the drug under investigation. Figure 6b was about the species distribution curve of the drug which give us information about drug species at studied pH range, this information very important to interpreting the removal of drug in different pH. The drug is strongly attached to the adsorbent due to electrostatic attraction and numerous hydrogen bonds, The drug is strongly attached to the adsorbent due to electrostatic attraction and numerous hydrogen bonds^50–53^.

#### The effect of dose of adsorbent

We tested various amounts of adsorbents, ranging from 0.0125 g to 0.2 g/50 mL of 50 µg/mL FLX. As shown in Fig. 6e), the removal efficiency increases as the dose of the adsorbent increases, reaching its highest value at 0.1 g LAC. After that, a steady stateoccurs due to the lack of additional active sites needed for higher adsorption capacity.

#### Effect of temperature on the adsorption process

**Figure 7:**
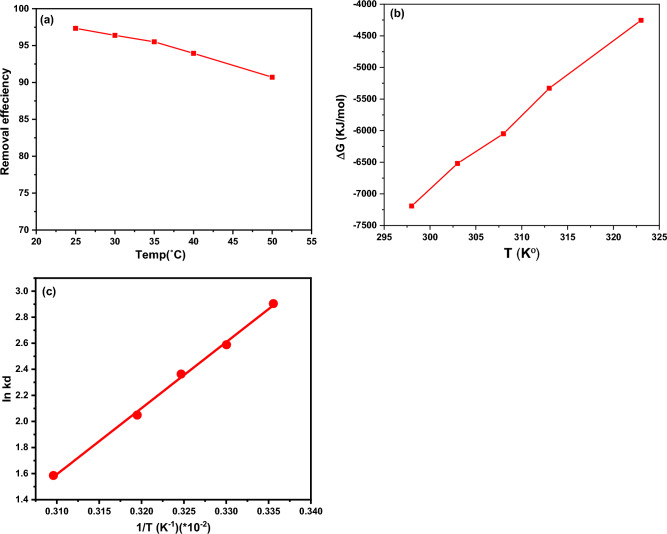
(**a**) The effect of temperature on the LAC's efficiency to remove FLX (**b**) a plot of Gibbs free energy change (G°) against temperature T (K) and (**c**) a plot of ln Kd against 1/T (K − 1).

The adsorption process was studied at temperatures of 25, 30, 35, 40, and 50 °C, with temperature being an important factor. In Fig. 7a, an inverse proportional relationship is shown between removal efficiency and temperature, indicating an exothermic physical adsorption process based on Le Chatelier's principle^40^. This relationship may be due to the weak binding between FLX and the synthesized adsorbent^54,55^. The results of the pre-screened tests in Table 2 were used to determine the Gibb free energy change (G°), enthalpy (H°), and entropy (S°), as well as the Kd = (qe/ce) values at different temperatures using the Van't Hoff Equation^56^.

3 $${\text{ln}}\;{\text{K}}_{{\text{d}}} = \Delta {\text{S}}^{^\circ } /{\text{R}}{-}\Delta {\text{H}}^{^\circ } /{\text{RT}}$$

Kd represents the equilibrium constant (L/mg), R is the gas rate constant (8.314 J/mol K), ∆H° is the change in adsorption enthalpy (kJ/mol), ∆S° is the adsorption entropy (derived from the intercept and slope of the straight-line plot of ln Kd vs 1/T (/K), and ΔG° is the Gibbs free energy that can be calculated using Eqs. (4, 5). The plot of ΔG° versus temperature is shown in Fig. 6b.

4 $$\Delta G^{^\circ } = - {\text{RT ln K}}_{{\text{d}}} = \Delta {\text{H}}^{^\circ } - {\text{T}}\Delta {\text{S}}^{^\circ }$$

5 $${\text{ln K}}_{{\text{d}}} = - \Delta {\text{H}}^{ \circ } /{\text{R}}\left( {{1}/{\text{T}}} \right) + \Delta {\text{S}}^{ \circ } /{\text{R}}$$

**Table 2:** The thermodynamic parameters of the adsorption process of FLX on LAC.

Material	T (K)	ΔG° (kJ/mol)	ΔH° (kJ/mol)	ΔS° (kJ/mol K)
LAC/AC	298	− 7192.48	− 62,874.29	− 197.5
	303	− 6518.13	− 62,874.29	− 197.5
	308	− 6049.51	− 62,874.29	− 197.5
	313	− 5329.11	− 62,874.29	− 197.5
	323	− 4254.97	− 62,874.29	− 197.5

The plot of ln Kd versus 1/T (K − 1) in Fig. 7c was linear, and the slope and intercept of the plot were used to determine the entropy change (ΔS°) and the enthalpy change (ΔH°). The Gibbs free energy (ΔG°) could be calculated using the Eq. (4). The negative values of ΔG°, H°, and S° indicate that the FLX adsorption process on LAC was carried out through a spontaneous exothermic process^57,58^.

#### Adsorption isotherm

The experimental data for FLX adsorption on LAC indicates that six isothermal nonlinear equilibrium models were used: Langmuir^59^, Freundlich (two parameters)^60^, Langmuir–Freundlich and Sips^61^, Redlich-Peterson (three parameters)^62^ and Baudu (four parameters)^63^. Modeling the adsorption experimental data is crucial for determining the extent of adsorption and optimizing the adsorption process. Langmuir isotherms are used for monolayer adsorption at homogeneous sites, while Freundlich isotherms are used for multilayer adsorption at heterogeneous sites. According to Ayawei et al.^64^, the Langmuir–Freundlich isotherm was created to capture heterogeneous surfaces. According to Koble and Corrigan^65^, it depicts the distribution of adsorption on an adsorbent surface. This isotherm becomes a Freundlich isotherm at low concentrations of adsorbate, and a Langmuir isotherm at high concentrations^64^. For both the surface complexation model and experimentally predicted datasets for the sorbent in use, the Langmuir–Freundlich isotherm model produced accurate predictions. The suggested analytical isotherm framework can aid in lowering computational demands, modelling complexity, and model development time.

**Figure 8:**
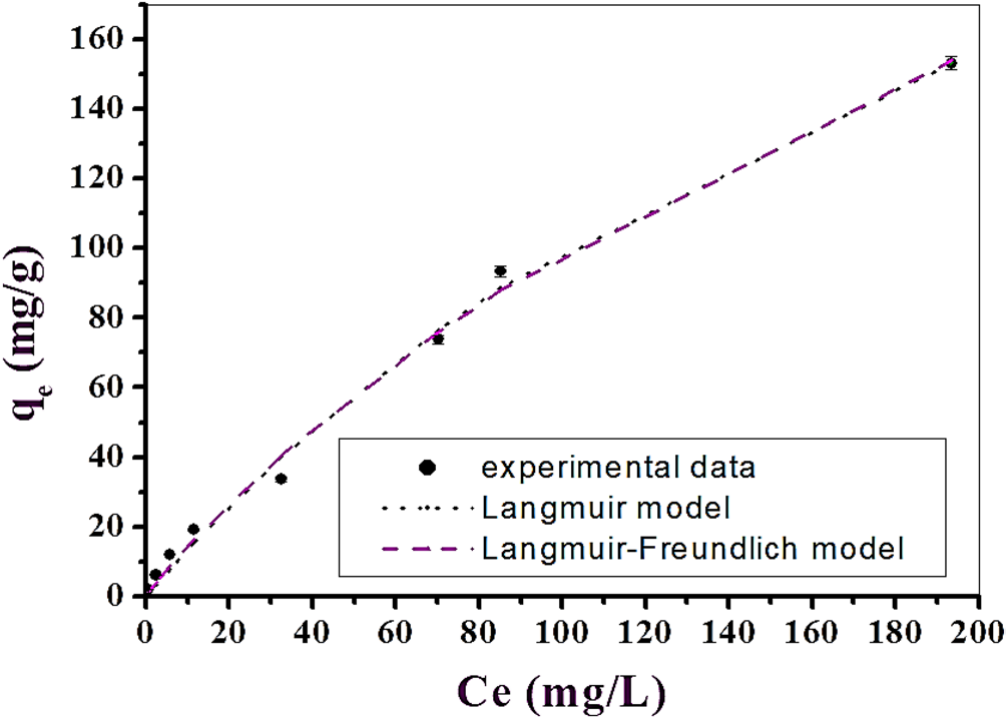
Experimental FLX-LAC adsorption isotherm data were fitted using representative two- and three-parameter isotherm models.

Overall, it was evident that the acquired experimental data of the adsorption process were fitted with two and three and four parameters models. The Langmuir–Freundlich model had the best fit (R^2^ = 0.99) and showed the highest adsorption capacities (qmax = 450.92 mg/g), as shown in Table 3. Based on this data, LAC can be considered an ideal adsorbent for the FLX adsorption process from aqueous solution, with a maximum adsorption capacity (qmax) of 450.92 mg/g, as shown in Fig. 8.

**Table 3:** The models for the LAC adsorption isotherm.

Isotherm models	Expression	Adjustable model parameters	Values	*R*^2^
Two-parameters isotherm				
Langmuir	$q_{e} = \frac{{q_{max} K_{L} C_{e} }}{{1 + K_{L} C_{e} }}$	q_max_	356.18	0.99
		*K*_L_	0.004	
Freundlich	q_e_ = *K*_f_ C_e_^1/n^_f_	*K*_f_	2.98	0.99
		1/*n*_f_	0.755	
Three-parameters isotherm				
Langmuir–Freundlich	$q_{e} = \frac{{q_{max} (K_{LF} C_{e} )^{{\beta_{LF} }} }}{{1 + K_{LF} C_{e} )^{{\beta_{LF} }} }}$	q_max_	450.92	0.99
		*K*_LF_	0.0025	
		β_LF_	0.93	
Sips	$q_{e} = \frac{{q_{max} K_{S} C_{e}^{{n_{s} }} }}{{1 + K_{S} C_{e}^{{n_{s} }} }}$	q_max_	350.66	0.97
		*K*_S_	0.0034	
		*n*_s_	1.04	
Redlich–Peterson	${q}_{e }=\frac{{K}_{RP} {C}_{e}}{1+ {a}_{RP}{C}_{e}^{{\beta }_{RP}}}$	${K}_{RP}$ ${a}_{RP}$ β_*RP*_	1.15 0 1.94	0.99
Four-parameters isotherm				
Baudu	$q_{e} = \frac{{q_{max} b_{o} C_{e}^{1 + x + y} }}{{1 + b_{o} C_{e}^{1 + x} }}$	q_max_	350.54	0.98
		*b*_o_	0.0034	
		X	0	
		Y	0.043

#### Adsorption kinetics

**Figure 9:**
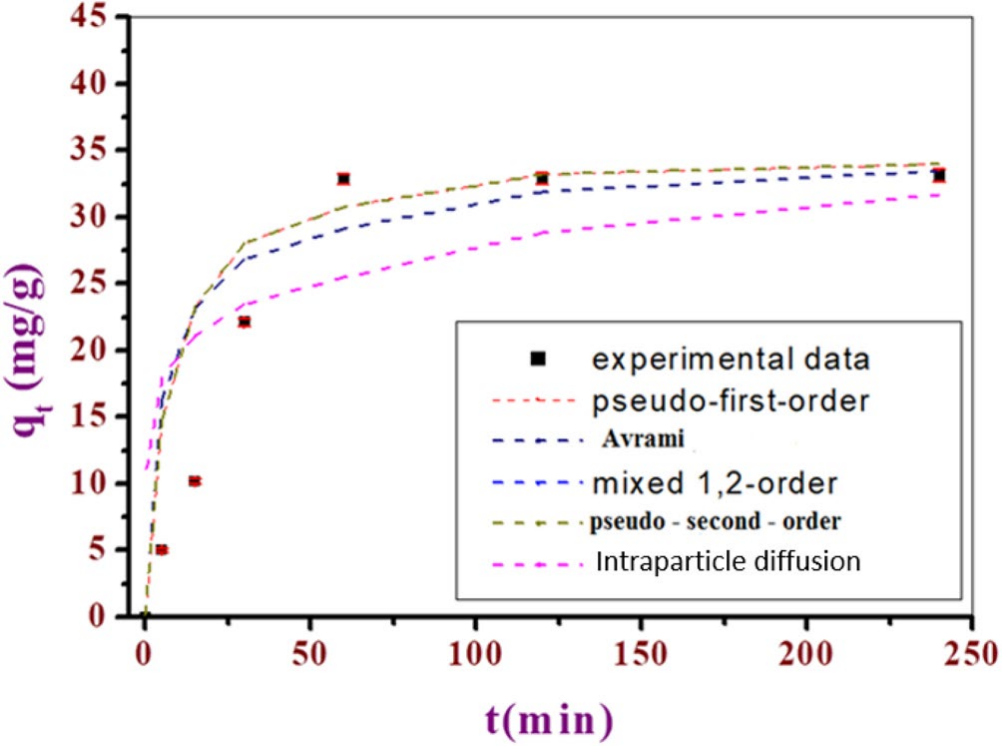
Experimental kinetic study and its fitting through applying different models of FLX adsorption on LAC.

Studying adsorption kinetics is important for understanding how quickly a substance is adsorbed onto an adsorbent's surface. This helps to determine the influence of different conditions on the speed of the process using models that describe the reaction. Additionally, adsorption kinetics help to determine the mechanism of dye adsorption onto the adsorbent material. Pesudo-first-order kinetic model describes the relationship between the change in time and the adsorption capacity with an order of one. Whereras, pseudo-second-order model explains how dissolved FLX ions are adsorbed on to the surface of the adsorbent through cation exchange or chemical bonding, indicating that a chemical process is involved in the adsorption. Mixed 1,2 order assesses the kinetics of dye adsorption onto mesoporous carbons from aqueous solution onto mesoporous carbons from aqueous solution. The Avrami's model to describe the kinetics of phase transformation under the assumption of spatially random nucleation has been used for assessing the adsorption of FLX from aqueous solution. The intraparticle diffusion describes the transportation of species from the bulk to solid phase of porous material in solution. The initial stages of the adsorption process on LAC showed rapid adsorption of FLX for up to 45 min, likely due to the presence of active sites. After this, the adsorption process slowed down due to a decrease in the number of active sites, reaching a steady state. The effect of time on removal efficiency was assessed using various models. The pseudo-second-order model has shown the highest adsorption capacity with best fit with a high R^2^ value of 0.99 which might be due to cation exchange or chemical bonding. The mixed 1,2-order, Avrami, and pseudo first order models also provided a good fit with less adsorption capacities, as shown in Table 4. On the other hand, The intraparticle diffusion model had a reasonable fit with an R^2^ value of 0.69, as depicted in Fig. 9.

**Table 4:** Coefficients of the pseudo-first-order, second-order adsorption kinetic, Avrami, and intraparticle diffusion models for adsorption of FLX by LAC.

Kinetic models	Equation	Parameters	LAC
Pseudo-first-order	q_t_ = qe (1 − e^−k^_1_^t^)	K_1_	0.037
		Q_e_	34.36
		R^2^	0.98
Pseudo-second-order	q_t_ = q_e_^2^k_2_t 1 + q_e_k_2_t	K_2_	0.0012
		Q_e_	39.32
		R^2^	0.99
Mixed 1,2 order	q_t_ = q_e_ (1 − exp(− kt)1 − f_2_exp(− kt)	K	0.04
		Q_e_	34.36
		F_2_	0
		R^2^	0.98
Avrami	q_t_ = q_e_[1 − exp(− k_av_t)^n^_av_]	Q_e_	34.01
		K_av_	0.40
		n_av_	0.1
		R^2^	0.99
Intraparticle diffusion	q_t_ = K_ip_√t + C_ip_	K_ip_	1.93
		C_ip_	10.5
		R^2^	0.69

#### *The FLX and protonated HFLX*^+^*molecular DFT calculations*

**Figure 10:**
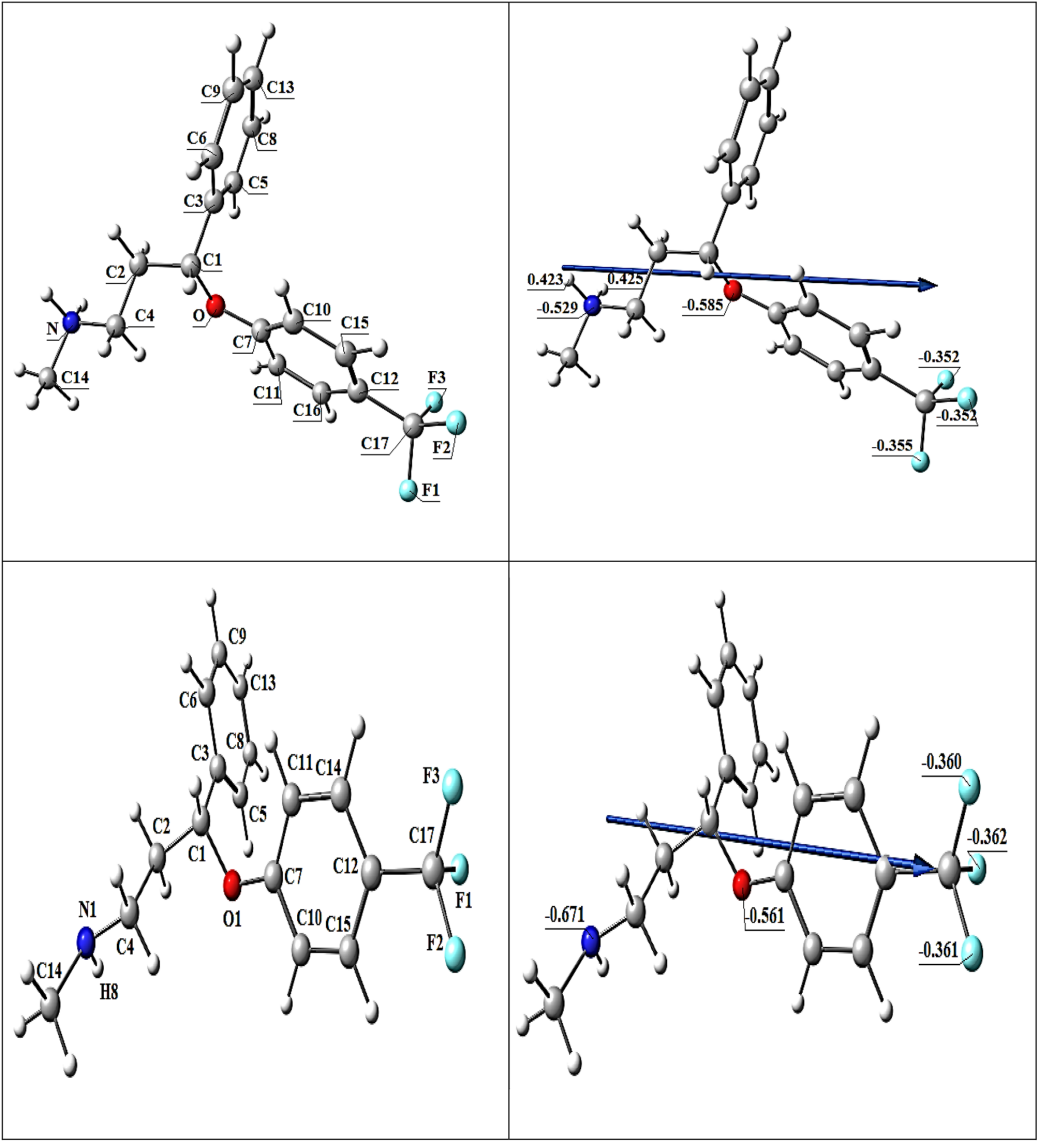
Optimized structure of FLX (upper) and HFLX^+^ (lower), and the of the dipole moment vector by DFT, B3LYP/6-311++ G (d, p).

Figure 10 displays the most stable low-energy configurations for FLX and HFLX^+^ as the optimal setups. The natural charges from NBO (Natural Bond Orbital Analysis) are presented for FLX (on nitrogen (− 0.671), oxygen (− 0.561) and fluorine (− 0.360, − 0.361 and − 0.362) atoms) and for HFLX + (on nitrogen (− 0.529), hydrogen (+ 0.423 and + 0.425), oxygen (− 0.585) and fluorine (− 0.352, − 0.352 and − 0.355) atoms). The molecular electrostatic potential (MEP) surfaces of FLX and HFLX^+^ are shown in Fig. 12, displaying positive (blue) and negative (red) areas.

Table 5 presents the measured total energy for FLX as well as the HOMO (highest occupied molecular orbital), LUMO (lowest unoccupied molecular orbital), and dipole moment. A larger negative value of the total energy of HFLX^+^ indicates higher stability. The energy gap (Eg) = ELUMO − EHOMO for FLX and HFLX^+^ are 5.3395 and 4.2477 Hartree, respectively, as shown in Table 5 and Fig. 13. There are numerous reactivity descriptors, including ionization potential (I), electronegativity (A), electron affinity (E), chemical potential (C), hardness (H), softness (S), and electrophilicity index (E), all of which are derived from HOMO and LUMO energies. These descriptors can be used to understand various reactivity-related aspects of chemical reactions (Table 5).

**Table 5:** Calculated total energy, ionization energy (I), electronegativity (χ), electron affinity (A), chemical hardness (η), global softness (S), chemical potential (μ), and electrophilicity index (ω) of FLX and HFLX^+^.

Property	FLX	HFLX^+^
The total energy E (a.u.)	− 1088.459	− 1088.830
HOMO (eV)	− 6.3613	− 9.2628
LUMO (eV)	− 1.0218	− 5.0151
E_g_ = E_LUMO_ − E_HOMO_ (eV)	5.3395	4.2477
Dipole moment (Debye)	3.9518	24.0474
Ionization potential I = − E_HOMO_	6.3613	9.2628
Electron affinity A = − E_LUMO_	1.0218	5.0151
Electronegativity χ = (I + A)/2	3.6916	7.1390
Chemical hardness η = (I − A)/2	2.6698	2.1239
chemical softness S = 1/2η	0.1873	0.2354
chemical potential μ = − χ	− 3.6916	− 7.1390
Electrophilicity ω = μ2/2η	2.5522	11.9981

**Figure 11:**
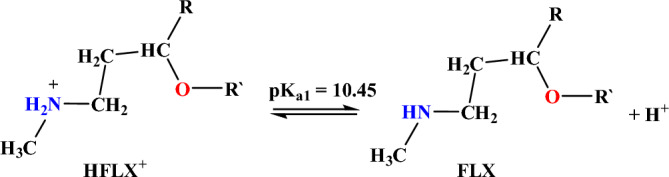
Acid–base equilibrium of protonated HFLX^+^, R, and R` are the remaining FLX molecule.

**Figure 12:**
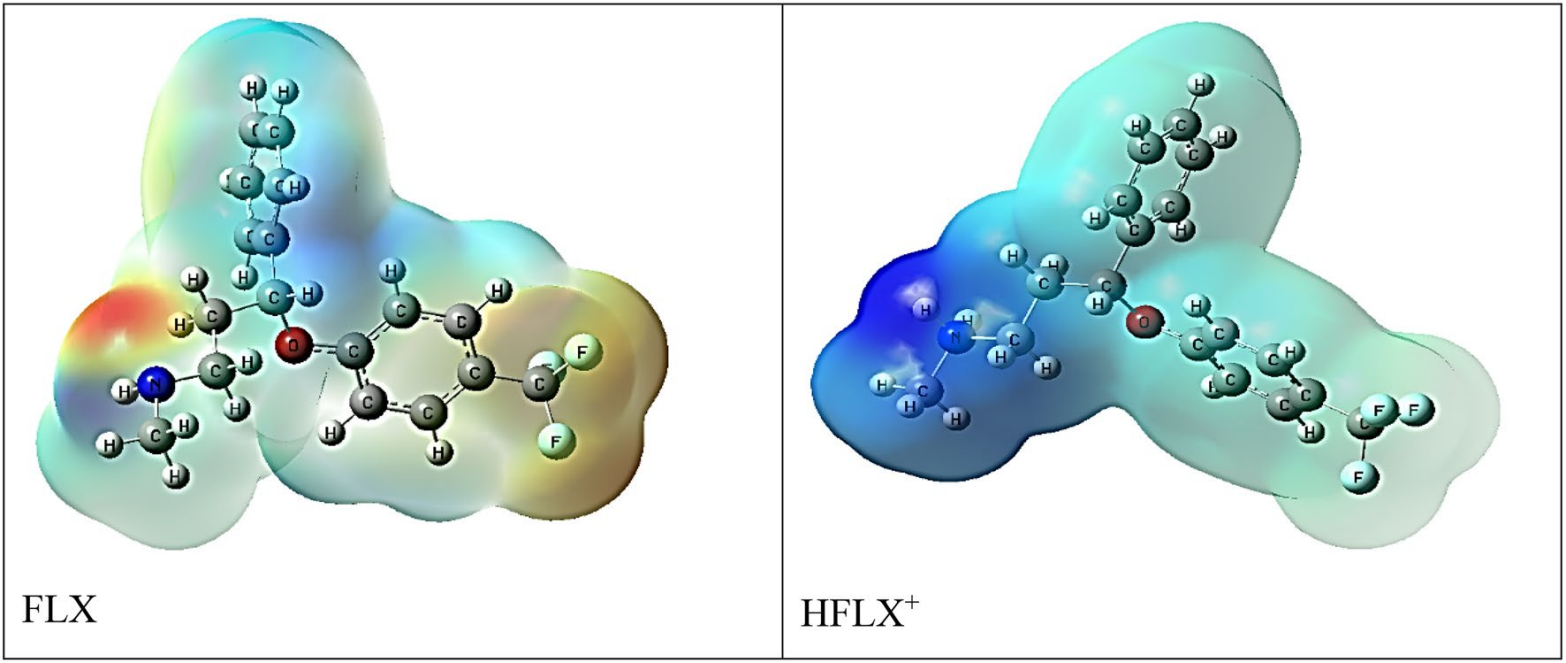
FLX and HFLX^+^ Molecular electrostatic potential (MEP) surface.

**Figure 13:**
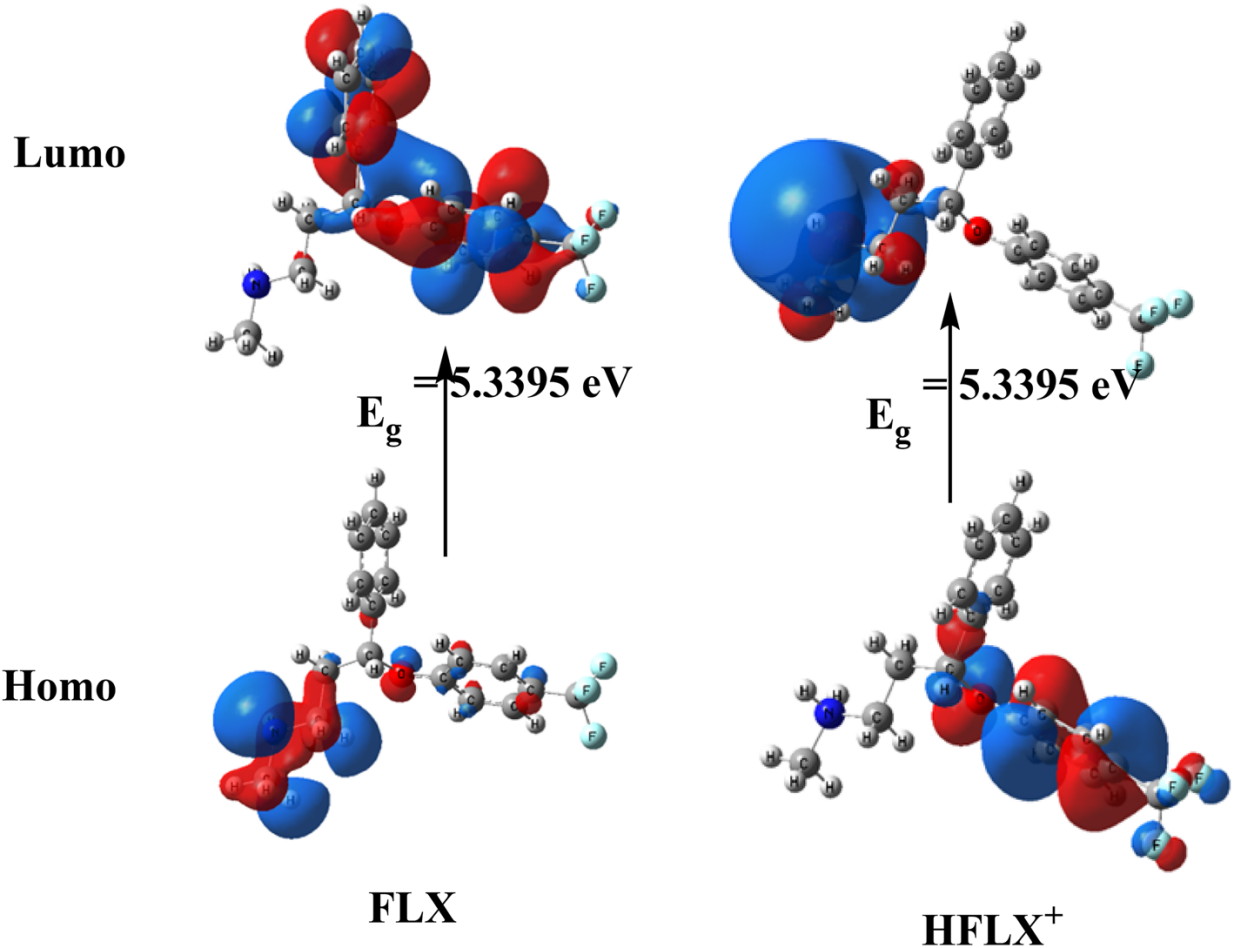
FLX and HFLX^+^ charge density maps in HOMO and LUMO.

Figure 11 presents the acid–base equilibrium for protonated (HFLX^+^), and Fig. 6c displays the titration curve to determine the dissociation constants of FLX and understand the concentration and form of each species at the optimal pH for maximum adsorption capacity. using a. At 25 °C and 0.1 M ionic strength, protonated nitrogen dissociates with a pKa value of 10.45. (Fig. 6b) displays a FLX species concentration distribution plot. At low pH levels up to pH 10.4, the protonated HFLX^+^ species predominate, followed by the species FLX, which starts after pH 8.3, and the unprotonated species FLX, which predominates after pH 10.40 (Figs. 12, 13).

#### Mechanism of adsorption

**Figure 14:**
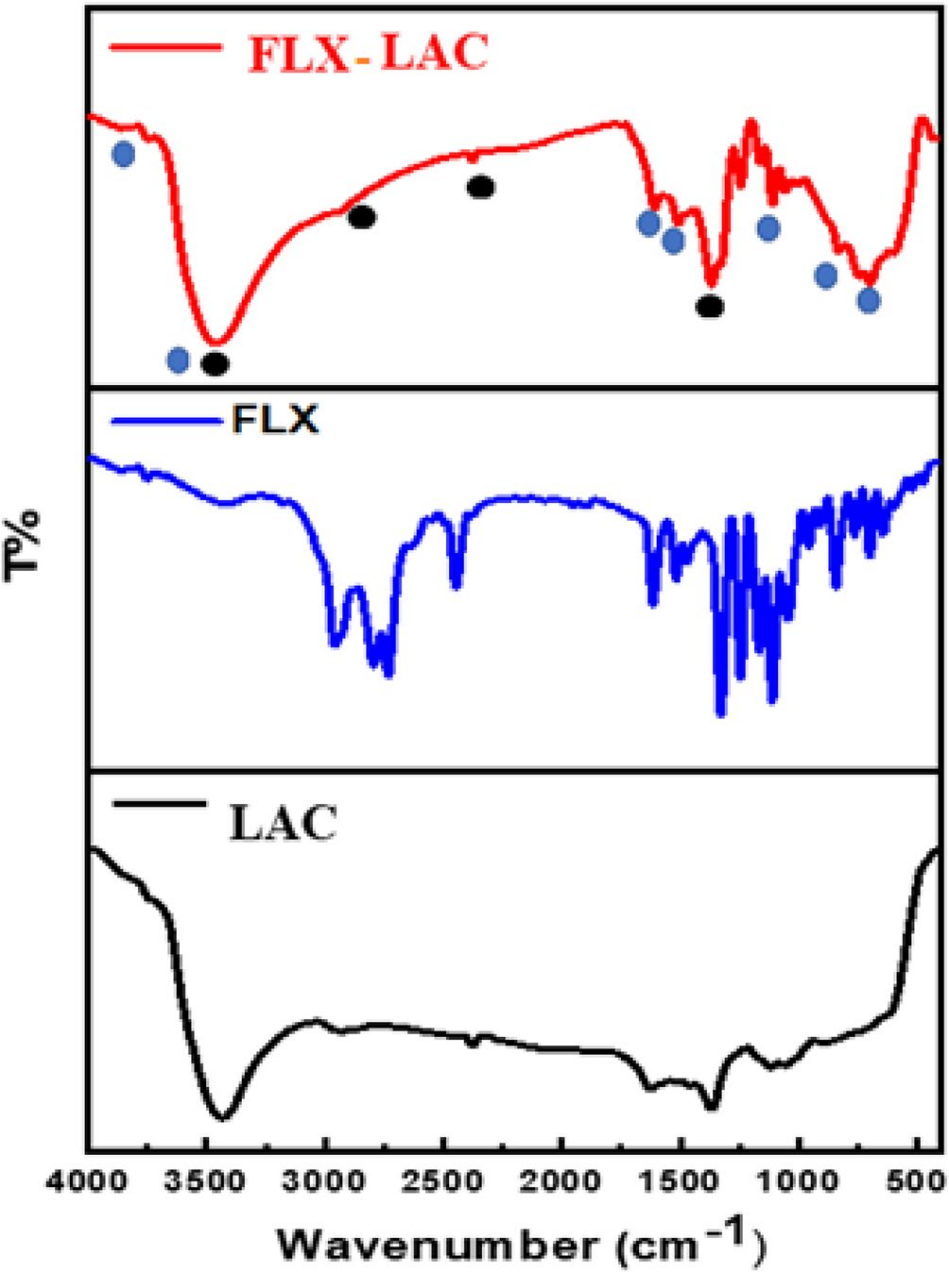
FTIR spectra of FLX- LAC, FLX and LAC.

The activated carbon surface forms hydrogen bonds with the hydroxyl groups of LAC and with the H-donors on the adsorbent surface sites and the H-acceptors through oxygen or nitrogen atoms of FLX These interactions, along with Van der Waals forces, contribute to the high chemical stability of the synthesis. At pH 10, surface diffusion, intra-particle diffusion, coordination interaction, hydrogen bonding, and electrostatic attraction can all occur, leading to the highest FLX removal percentage at that pH^50,66^. The core Al, Zn, or Co metals in the LAC attract atoms (O and N) with lone pair electrons in FLX adsorption. As appearing in the FTIR of FLX spectrum (Fig. 14), revealed distinct bands at 1400–1000 cm^−1^ for the C-halogen group (C–F) and 1294.5 cm^−1^ for the amine group (C–N). On the other hand, the peak at 2000–1650 cm^−1^ is assigned to C–H of the aromatic chain. The band at 1518.2 cm^−1^ belongs to the C=C stretching vibrations. The phenoxy stretching vibration (C–O-Aromatic group led to the peak at 1250–1200 cm^−1^^35^. The peak that appeared between 2800 and 3076 cm^−1^ is related to the presence of the aromatic chain. The bands at 2960 and 2850 cm^−1^ were also assigned to the C–H vibrations^15,67^. Furthermore, N–H group stretching vibration was detected at 3421.44 cm^−1^. The FLX-LAC spectrum revealed an intense broad peak at 3447.03 cm, which is attributed to O–H stretching vibrations caused by water molecules physisorption and interlayer hydrogen bonds. The successful loading of FLX on the surface of LAC is confirmed by changes in the FTIR spectrum. The appearance of Fluoxetine functional groups after its adsorption by LAC is indicated by distinct peaks at 1516 cm^−1^ (C=C), 1324 cm^−1^ (C–F) stretching vibrations. At around 692 cm^−1^ is a suggestive peak of mono-substituted FLX phenyl ring vibrations^15^.

### In-Vivo study experimental animals

Sleep is a natural state of rest where consciousness decreases and the body's activity and reaction to external stimuli also decrease. Sleep deprivation can impact physiology and is linked to various health issues such as obesity, diabetes, hypertension, anxiety, depression, and neurodegenerative diseases like Alzheimer's^68^. Sleep is crucial for the body's recovery, maintaining a healthy immune system, preventing sleep-related diseases, and supporting memory consolidation and learning processes in the brain^69^ and potential for learning^70^.

Intermittent sleep is a common issue for people with depression, and it is a diagnostic criterion for the disease. Insomnia in the middle of the night is a key symptom of depression, and all antidepressants aim to improve sleep. However, some antidepressants may initially disrupt sleep, while others can lead to long-term problems due to over-sedation. The best results for promoting sleep are often achieved with a modest dose given early enough before bedtime, as part of complex cognitive-behavioral protocols for reating insomnia^71,72^. The impact of antidepressants on sleep is often overlooked, but it can significantly affect treatment outcomes and compliance. Many side effects of antidepressants and persistent symptoms are related to sleep.

After being orally administered, the acute toxicity of FLX-LAC, FLX, and LAC was tested in rats (Table 6). Poisoning indicators included tremors, rapid breathing, arched back, convulsions,unconsciousness, and eventual death. The probability of death began to increase at 185 mg/kg body weight for FLX-LAC, compared to 220 mg/kg body weight for FLX. The LD_50_ for FLX-LAC and FLX was determined to be 368 mg/kg and 410 mg/kg, respectively, with LD_100_ achieved at 750 mg/kg and 720 mg/kg, as shown in Tables 7 and 8. These results indicate that both FLX-LAC and FLX are safe for use in pharmacological studies. In this study, LD50 values of 19.3 mg/kg and 7 mg/kg were used to determine hypnotic activity for the duration of the sleep period. The estimated therapeutic dose at 1/20-th was 9 mg/kg for LAC, compared to an LD_50_ of 181 mg/kg for LAC.

**Table 6:** FLX-LAC, FLX, and LAC doses with total number of animals tested and mortality rates.

Group	Dose (mg/kg b.wt.)	No. of animals/group	No. of dead animals
FLX-LAC	50	10	0
	100	10	0
	150	10	0
	200	10	1
	250	10	1
	300	10	1
	350	10	2
	400	10	4
	450	10	7
	500	10	8
	550	10	10
	600	10	10
FLX	50	10	0
	100	10	0
	150	10	0
	200	10	0
	250	10	1
	300	10	1
	350	10	2
	400	10	3
	450	10	5
	500	10	8
	550	10	10
	600	10	10
LAC	100	10	1
	200	10	2
	300	10	5
	400	10	7
	500	10	10
	600	10	10

**Table 7:** LD_50_ and LD_90_ estimation of FLX-LAC, FLX and LAC.

Treatment	LD_50_ (%) (LC_50_)	95% CL		LD_90_ or LC_50_	95% CL		X^2^ (df = 11)	P*
		LCL	UCL		LCL	UCL		
FLX-LAC	386	349	422	550	492	659	14.13	0.22
FLX	410	376	444	549	499	546	8.8	0.636
LAC	181.03	126.57	230.49	363.60	279.04	631.87	2.691 df = 3	0.442

**Table 8:** Probit analysis comparison of LD_0_, LD_20_, LD_50_, LD_90_, and LD_100_ values for FLX-LAC, FLX, and LAC materials.

FLX-LAC (mg/kg)	Miller-Tainter's method
LD_0_	185
LD_20_	217
LD_50_	386
LD_90_	550
LD_100_	750
FLX	
LD_0_	220
LD_20_	256
LD_50_	410
LD_90_	594
LD_100_	720
LAC	
LD_0_	45
LD_20_	59
LD_50_	181
LD_90_	363
LD_100_	650

**Figure 15:**
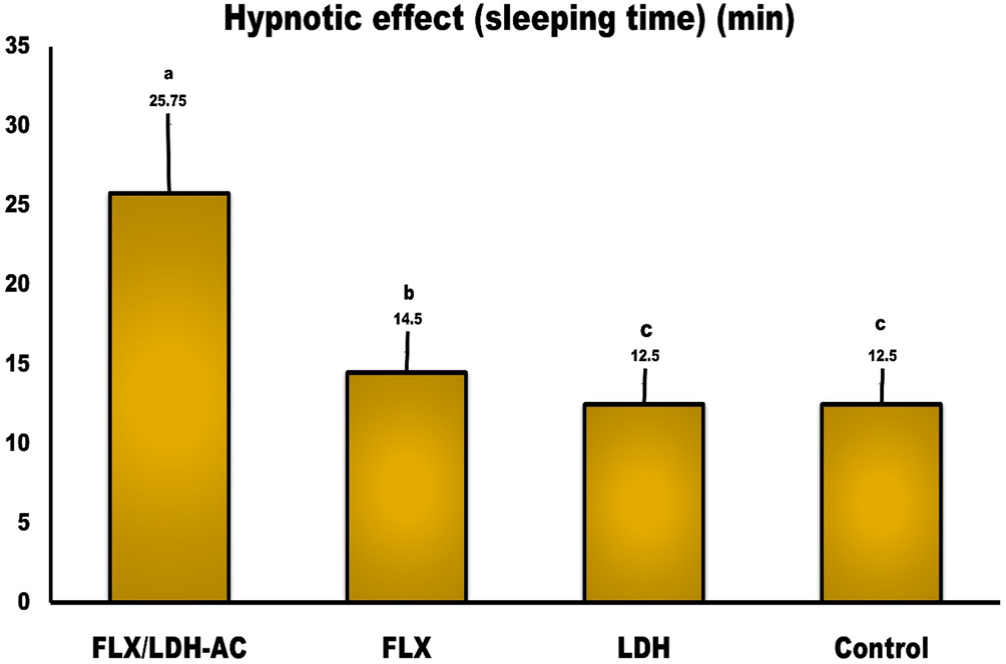
Sleeping time (Hypnotic effect) of FLX-LAC, FLX and LAC in rats. (n = 10) Implies plus the SD.

Toxicity increased with higher drug doses, and sleep time prolongation was observed for the different materials tested. Pretreatment with FLX-LAC, FLX, and LAC at dose levels of 19.3 mg/kg, 7 mg/kg and 9 mg/kg body weight, orally, before the use of thiopental sodium to induce sleep, significantly increased sleep time compared to the negative control group (Fig. 13). Pretreatment with FLX-LAC also significantly doubled the time to fall asleep (25.7 min) compared to FLX alone (14.5 min), indicating that FLX was released from the LAC layers, resulting in a longer effect. Non-significant activity was observed in LAC and untreated control negative rats during sleep time (12.5 min), as shown in Fig. 15.

LCL, UCL, X^2^, df, and chi-square are abbreviations for lower and upper confidentiality limits, respectively. In lethal amounts, 50% and 90% of the population dies, respectively. p > 0.05 is not significant.

The LD50 was calculated using probit analysis in this research. The therapeutic doses for the gastroprotective and hypnotic study were determined to be 1/20 of the estimated LD_50_:

$$\begin{aligned} & {\text{FLX - LAC}}\;{\text{LD}}_{50} = 386 \times 1/20 = 19.3\;{\text{mg/kg}}. \\ & {\text{FLXLD}}_{50} = 410 \times 1/20 = 7\;{\text{mg/kg}}. \\ & {\text{LACLD}}_{50} = 181 \times 1/20 = 9\;{\text{mg/kg}}. \\ \end{aligned}$$

The research aimed to explore the potential of extending sleep duration as a treatment for sleep problems, particularly in individuals with depression. The focus was also on developing a safe formula using activated carbon, which is crucial for absorbing toxins and gases, reducing pain, and preventing brain inflammation, all of which contribute to improved sleep. The use ofactivated carbon in the synthesis recipe was intended to eliminate gases and toxins from the digestive system, promoting relaxation and faster sleep onset besides also its effects the gut microbiota had been investigated^73,74^. Many doctors recommend using activated carbon before bed to prevent brain inflammation caused by toxins, which can disrupt sleep in addition to its use in treatment of intoxication^75,76^. Inflammatory overload can lead to persistent fatigue and difficulty falling asleep, with bad gut bacteria being a source of inflammatory toxins. A recent study found that the Western diet is the main cause of Irritable Bowel Disease, indicating that the foods we consume significantly impact inflammation levels in the body. Consuming the typical American diet increases the likelihood of suffering from bad gut bacteria and inflammation. Activated carbon acts as a vacuum in the digestive system, collecting toxins before they enter the body and protecting the brain from potential infections. Better sleep leads to reduced inflammation in the brain^77^.

Drug delivery systems have improved the therapeutic efficacy and side effects of systemic drugs. There is a lack of research on how fluoxetine with activated carbon affects sleep time.

Layered double hydroxide (LDH) is being explored as a new method for pharmaceutical delivery^78,79^ due to its safety and low toxicity^80^. Nanotechnology has proven effective in treating sleep problems by allowing continuous and controlled drug delivery^81^.

The size and integration of nanocarriers in layers have a significant impact on pharmacokinetics and pharmacodynamics^82^. Nanoparticles enhance the effect of carrier molecules, such as pharmaceuticals, due to their higher surface-to-volume ratio^83^. Coatings of LDH effectively increase the duration of action of fluoxetine. The cobalt, zinc, and aluminum ions of LDH have antibacterial properties and act as scavengers for free radicals in the presence of reactive oxygen species (ROS). Additionally, Co, Zn, and Al help achieve a high level of activity in a shorter period of time^84^. Nanoparticles containing antidepressant drugs are effective in treating brain diseases and infections due to their small size^85^. efficient adherence, and ability to travel across the blood–brain barrier. Zinc is believed to be involved in a wide range of biological processes at a molecular and physiological level, and recent research suggests that it may also play a role in regulating sleep. According to a new study, serum zinc content fluctuates with the amount of sleep a person gets, and taking zinc by mouth improved the quantity and quality of sleep in mice and people. Zinc supplements also increase the duration of sleep, which can help improve sleep quality. Since the discovery of zinc's important role in regulating basic activities such as memory and now sleep, its location in the central nervous system has become more important^86^. Cobalt was found in the white matter of the brain, specifically in the corpus callosum. This area showed higher endoplasmic reticulum stress, fewer myelin-binding proteins, disorganized myelin sheaths, and worse axon profiles compared to the rest of the brain. Cobalt is an essential component of vitamin B_12_, which plays a role in neurological diseases^87^. Layered hydroxides are innovative nanocarriers for cellular drug delivery and also aid in the action of antibiotics and medications. Their surface modification, due to good ion exchange capabilities, improves cellular drug delivery. Positively charged hydroxide layers enhance the penetration of cells and improve drug distribution by incorporating anionic pharmaceuticals into the layered hydroxides^88–90^. The cellular uptake of layered hydroxides decreases as the particle size increases, but the retention time mechanism ensures complete cellular uptake within 15 min^89,91,92^. Layered hydroxides can adsorb negatively charged drugs without the need for modification or functionalization due to their net positive charge. This has allowed for the conjugation and delivery of several negatively charged cytotoxic drugs into cells via regulated release^93,94^.

## Conclusion

Previous research has shown that using ternary LAC is more effective than other adsorbents for removing Fluoxetine HCL (FLX) residues from water. This is due to its low cost, ease of operation, and large surface area. The adsorption process has been sudied extensively, with factors such as pH, adsorbent dose, temperature, and FLX concentration found to have an impact. The optimal conditions for the adsorption process were found to be a pH of 10, an adsorbent dose of 0.1 g, and a temperature of 25 °C. The synthesized nanocomposite was also characterized using various techniques. Six nonlinear equilibrium models were tested, with a maximum adsorption capacity (q max) of 450.92 mg/g. Kinetic studies were conducted, and the safety and toxicity of the synthesized nanocomposite were examined, confirming its safety and its potential role in inducing anesthesia and promoting sleep in a rat model.

## Data Availability

The datasets used and/or analysed during the current study available from the corresponding author on reasonable request.

## Abbreviations

q_t_, q_e_: The (mg/g) adsorption capacities at time t (min) and equilibrium
k_1_: The rate constant pseudo-first-order (/min)
k_2_: The rate constant pseudo-second-order (/min)
f_2_: The mixed 1,2 order coefficient (dimensionless)
k , k_ip_: The measurement of the diffusion coefficient (mg/g min (1/2)) and the adsorption rate constant (mg/g/min) respectively.
c_ip_: The intraparticle diffusion constant (mg/g)
k_av_: The Avrami rate constant (/min)
n_av_: The Avrami component (dimensionless).
FLX: Fluoxetine
LDH: Layered double hydroxide
LAC: Layered double hydroxide-activated carbon
FLX-LAC: Fluoxetine-layered double hydroxide-activated carbon (formed nanocomposite)
